# The Rheology of PEOT/PBT Block Copolymers in the Melt State and in the Thermally-Induced Sol/Gel Transition Implications on the 3D-Printing Bio-Scaffold Process

**DOI:** 10.3390/ma12020226

**Published:** 2019-01-10

**Authors:** Veronica Vanzanella, Marco Scatto, Erwin Zant, Michele Sisani, Maria Bastianini, Nino Grizzuti

**Affiliations:** 1 Dipartimento di Ingegneria Chimica, dei Materiali e della Produzione Industriale, Università degli Studi di Napoli Federico II, Piazzale V. Tecchio 80, 80125 Napoli, Italy; veronica.vanzanella@unina.it; 2 Nadir S.r.l., c/o Scientific Campus University Ca’ Foscari Venezia, Via Torino 155b, 30172 Mestre, Italy; scattom@gmail.com; 3 PolyVation b.v., Kadijk 7d, 9747AT Groningen, The Netherlands; E.zant@polyvation.com; 4 Prolabin & Tefarm S.r.l., Via dell’Acciaio 9, 06134 Perugia, Italy; michele.sisani@prolabintefarm.com (M.S.); maria.bastianini@prolabintefarm.com (M.B.)

**Keywords:** poly(ethylene oxide terephthalate), poly(butylene terephthalate), random block copolymers, microphase separation, rheology, viscoelasticity, 3D-printing, scaffolds

## Abstract

Poly(ethylene oxide terephthalate)/poly(butylene terephthalate) (PEOT/PBT) segmented block copolymers are widely used for the manufacturing of 3D-printed bio-scaffolds, due to a combination of several properties, such as cell viability, bio-compatibility, and bio-degradability. Furthermore, they are characterized by a relatively low viscosity at high temperatures, which is desired during the injection stages of the printing process. At the same time, the microphase separated morphology generated by the demixing of hard and soft segments at intermediate temperatures allows for a quick transition from a liquid-like to a solid-like behavior, thus favoring the shaping and the dimensional stability of the scaffold. In this work, for the first time, the rheology of a commercial PEOT/PBT material is studied over a wide range of temperatures encompassing both the melt state and the phase transition regime. Non-isothermal viscoelastic measurements under oscillatory shear flow allow for a quantitative determination of the material processability in the melt state. Additionally, isothermal experiments below the order–disorder temperature are used to determine the temperature dependence of the phase transition kinetics. The importance of the rheological characterization when designing the 3D-printing scaffold process is also discussed.

## 1. Introduction

Poly(ethylene oxide terephthalate)/poly(butylene terephthalate) (PEOT/PBT) segmented block copolymers have been known for more than thirty years as bio-compatible materials [1]. Today, PEOT/PBT (or PEGT/PBT, from poly(ethylene glycol terephthalate), which is often used as a synonym name) is used in many bio-medical applications, such as drug release systems [2,3], dermal substitutes [4], bone fillers [5], nerve regeneration [6] and, most of all, scaffold manufacturing [7,8,9,10,11,12,13,14,15,16].

One popular technology for scaffold fabrication is the 3D-printing fused filament technology, where the scaffold is built by extruding a molten polymer thread on superimposed layers, according to a well-defined pattern [7,10,12,13]. The polymer is injected when in the liquid state, and then solidifies after the filament has been deposited onto the growing layers, thus originating the three-dimensional scaffold geometry.

A polymer suitable for bio-scaffolds must possess some obvious requirements. It must be bio-compatible and, when required, bio-degradable or bio-resorbable. Above all, it must be able to host a bio-instructive system, that is, the chemical and physical microenvironment required for cell viability, differentiation and growth [17].

Apart from the necessary bio-related properties, the polymer must exhibit a low melt viscosity, to allow for an easy injectability and filament production. Furthermore, the solidification kinetics must compromise between two simultaneous requirements: on the one hand, the necessity to quickly impart a sufficient consistency to allow for the scaffold to retain its shape and dimension. On the other hand, the somewhat opposite need to keep a liquid-like behavior for a sufficient time, in order to guarantee an optimal healing between two consecutive filament layers. The material rheological behavior in the melt state and during its phase transition stages, therefore, is crucial in determining the best manufacturing conditions.

Segmented copolymers such as PEOT/PBT are constituted by two segments with very different characteristics: the hard segment (HS, in this case PBT) and the soft segment (SS, PEOT). At high temperatures, the segments are well mixed, as the disordering Brownian effects prevail over the intersegmental interactions. As the temperature is lowered, HS and SS recognize their molecular incompatibility, which results in the formation of microphase-separated domains of soft and hard segments. The typical morphology is made of amorphous or semi-crystalline HS domains dispersed into a SS matrix. The characteristics of the microphase-separated morphology, which are mainly controlled by the chemistry and the relative composition of the two segments, crucially affect the mechanical and rheological properties of segmented copolymers [18].

While several studies deal with some relevant physical attributes of PEOT/PBT block copolymers, such as thermal, degradation, mechanical, and tribological properties [19,20,21,22,23,24,25,26], very little is known about their rheological behavior and its consequences on the 3D-printing process. In this paper, to our knowledge for the first time, the rheology of a PEOT/PBT polymeric material is experimentally studied in detail. Small amplitude oscillatory shear (SAOS) measurements are used to assess the temperature dependence of viscosity in the melt phase, and to investigate the subsequent cooling stages. In particular, the order–disorder transition (ODT) temperature, marking the onset of the microphase separation as well as the kinetics of the sol/gel process quantitatively determined. The experimental results are discussed also in terms of their consequences on the processing behavior during the 3D-printing stages for the manufacturing of bio-scaffolds.

## 2. Materials and Methods

PEOT/PBT was provided by PolyVation B.V. (Groningen, The Netherlands) and used as received. The material was labeled 300PEOT55PBT45, indicating a molar mass of the PEO segment of 300 Da and a 55/45 weight percent ratio between PEOT soft and PBT hard segments.

Differential scanning calorimetry (DSC) measurements have been performed on a Q2000 DSC apparatus (TA Instruments Inc., Pittsburgh, PA, USA). Temperature scans were performed at the standard heating/cooling rate of 10 °C/min. The first heating scan, from ambient temperature to +200 °C, was used to erase any thermal memory.

Rheological measurements have been conducted on an advanced rheometric extended system (ARES, Rheometrics Inc., Piscataway, NJ, USA) rheometer, equipped with a 0.2/2.0 Nm force rebalance transducer. SAOS measurements were performed using a parallel plate geometry with plates of 8 and 25 mm diameter. The amplitude was always chosen and, when necessary, changed in the course of the experiment in order to keep the material response within the linear viscoelastic limit. A 1 mm gap thickness was used and was kept constant in the non-isothermal tests by accounting for the tool thermal expansion. When necessary, the auto-tension feature was used to avoid loss of contact and minimize wall slip at the plate surface. The temperature control was guaranteed by a convection oven with an accuracy of ±1 °C. Fresh samples were used for each test. Control measurements performed at the beginning and at the end of each run confirmed the absence of thermal degradation.

All rheological measurements were performed on triplicate by using a fresh sample each time. No relevant variations, both qualitative and quantitative, were found between the different runs.

## 3. Experimental Results and Discussion

### 3.1. Calorimetry

**Figure 1:**
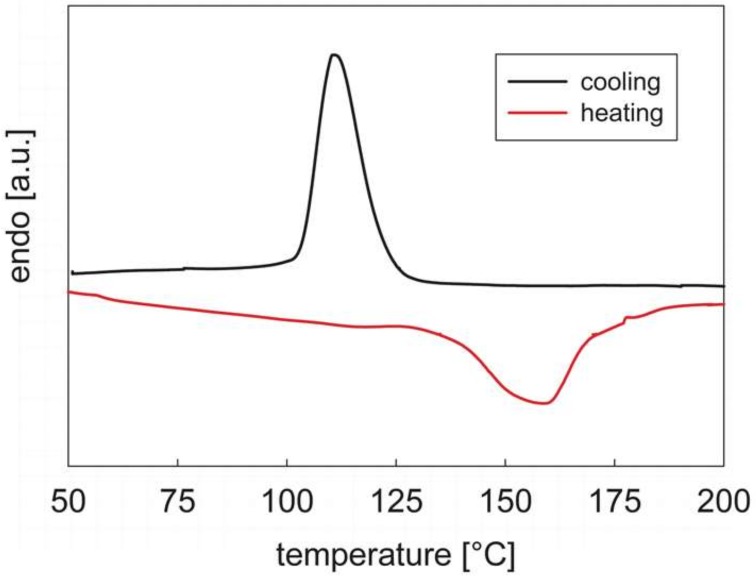
The DSC thermogram of PEOT/PBT between +50 and +200 °C. The cooling/heating rate is 10 °C/min. The cooling after the first heating and the second heating curves are shown.

Figure 1 shows the cooling and second heating DSC traces of PEOT/PBT between the temperatures of +50 and +200 °C (the interval of interest in this work). The sample shows a well-defined crystallization peak at 111 °C, probably corresponding to the crystallization of HS domains. Upon heating the endotherm signal is distributed over a much wider range of temperatures, although a clear melting peak is observed at 159 °C. The results of Figure 1 are compatible with those already available in the literature for similar PEOT/PBT samples [22,24].

### 3.2. Non-Isothermal Rheology

Rheological temperature ramps were performed under conditions similar to those used in calorimetric measurement. The sample is loaded at a temperature of 200 °C and, after thermal equilibration, is cooled at a fixed rate down to a temperature of 60 °C while measuring the linear viscoelastic moduli at the fixed frequency of 10 rad/s. Lower temperatures were out of range of the rheometer due to the increasing rigidity of the material. After an isothermal resting at 60 °C the temperature was increased again to the maximum value of 200 °C.

**Figure 2:**
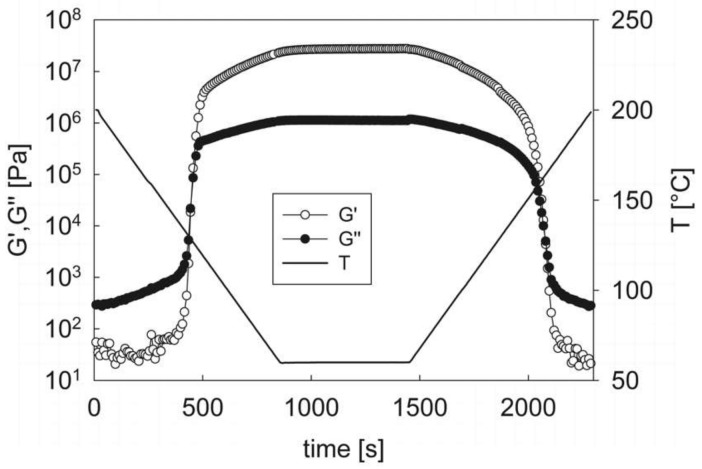
The elastic and loss moduli as a function of time during a cooling/heating ramp at 10 °C/min. The temperature history is reported on the right *y*-axis. The oscillation frequency is 10 rad/s. The strain is adjusted to guarantee the linear viscoelastic response at all times.

Figure 2 shows the results of the non-isothermal ramp for a cooling/heating rate of 10 °C/min. During cooling, three distinct regions of behavior can be clearly observed. At high temperatures, between about 200 and 140 °C, both moduli slowly increase upon decreasing temperature. On further cooling, a sharp increase of the moduli is observed over a very narrow temperature range. *G*′ grows faster than *G*″ in this region, and a cross-over between the moduli is observed at a temperature of about 128 °C. Starting from about 115 °C the moduli still increase upon cooling but to a much lesser extent than in the previous region, eventually tending to a flat plateau as the temperature approaches the final value of 60 °C. The material response is qualitatively symmetrical in the heating part of the experiment: the moduli first slowly decrease upon increasing temperature, then abruptly drop at intermediate temperatures, and eventually keep decreasing at a lower rate upon further increasing the temperature. The similarity is only qualitative, however, as the characteristic temperatures during heating are higher than those of the cooling phase. In any case, at high temperatures the rheological response becomes also quantitatively identical to that observed in the initial cooling stages, indicating that no thermal degradation has taken place and that the polymer has identically returned to its initial molten state.

The three-region viscoelastic behavior observed during the cooling and heating ramps can be directly related to the morphological evolution of the polymer microstructure. Considering the cooling section, the response in the high temperature region is typical of a homogeneous polymer melt. Here, as expected, the viscoelastic properties smoothly change with temperature. It can be noticed that the loss modulus is about one order of magnitude larger than the elastic modulus, thus indicating an almost purely viscous behavior of the material. The lack of a significant elastic component is also confirmed by the large scattering of the *G*′ data, as they fall around (or even below) the lower limit of the instrument sensitivity.

In the intermediate temperature range the dramatic change of the moduli is directly connected to the microphase separation between soft and hard segments. As the temperature decreases, the negative interactions between soft segments and hard ones prevail over the disordering effect of thermal motion. SS and HS domains become larger and possibly more ordered, thus determining the transition from a liquid-like to a solid-like rheological behavior, generally termed as the sol/gel transition. The point where the transition takes place is the so-called critical gel point [27], where the mobility of the polymer chains is essentially frozen by the growing microdomains. Although a rigorous definition of the critical gel point would require an analysis of the frequency response of the material [27], it is a generally well-accepted empirical rule to locate the critical gel point at the cross-over between the viscoelastic moduli [28]. In the case of Figure 2, therefore, the sol/gel transition is estimated to take place at a temperature of about 128 °C. This is a relevant processing information, in particular when the PEOT/PBT block copolymer is used for the manufacture of 3D-printed scaffolds. The sol/gel transition corresponds to the point where the material has solidified, and no further change in shape is possible.

In the final part of the cooling ramp the microdomains are now well-formed and the material has reached an essentially stable microstructure, as confirmed by the much lower increase of the moduli as compared to the sol/gel transition region. A closer inspection of the experimental data, however, indicates that kinetics effects are still present, and some additional time is required to reach equilibrium conditions. While the loss modulus remains constant at the final constant temperature of 60 °C, the elastic modulus still increases with time. The total change of about 20% reflects an increase of rigidity of the material. This “aging” phenomenon can be probably related to a continuous refinement and ordering of the hard segment microphase, as it has been already observed for other segmented block copolymers [29].

The same considerations made above for the cooling part of the experiment can be repeated for the heating section. At low temperatures, heating triggers the melting of the microdomains. Then, a progressive erosion of the hard phase domains takes place without substantial loss of connectivity, as already observed in the case of Thermoplastic Polyurethanes (TPU) [29,30]. Eventually, total melting occurs and the homogeneous liquid state is regained at higher temperatures.

**Figure 3:**
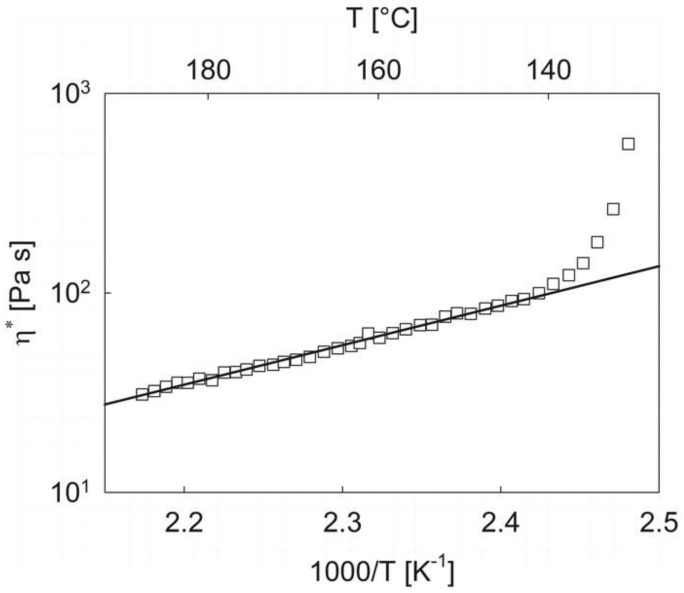
The logarithm of the complex viscosity as a function of the inverse absolute temperature in the early stages of the cooling ramp. The frequency is 10 rad/s. The straight line is the linear regression of the data before the jump due to the onset of the sol/gel transition (see Equation (1)). The linear temperature scale (in °C units) is reported on the top *x*-axis to facilitate the reading.

The cooling part of the viscoelastic response provides another technologically relevant piece of information. Figure 3 shows the temperature dependence of the complex viscosity of PEOT/PBT in the high temperature region.

When the log of viscosity is plotted as a function of the inverse absolute temperature, a linear behavior is observed down to about 140 °C, where the viscosity sharply increases due to the beginning of the sol/gel transition. At high temperatures, therefore, the rheological behavior is that of a purely viscous fluid whose viscosity follows a typical pseudo-Arrhenius dependence upon temperature. Separate experiments at a number of selected temperatures above the transition (not reported here for brevity) also show that the viscosity is not dependent upon frequency. It can be concluded that in the melt state the PEOT/PBT sample investigated in this work shows a purely viscous, Newtonian response whose temperature dependence, as obtained from a linear regression of the data of Figure 3, can be expressed in the following form:

(1) $$\eta \left(T\right)=\left(1.52\times 10^{-3}\right)\times 10^{\frac{1980}{T}}$$

where *T* is the absolute temperature (K) and the viscosity is in SI Units (kg m^−1^ s^−1^). Equation (1) quantitatively determines the viscous behavior of the melt. It is particularly useful, therefore, to optimize the processing parameters of the extrusion 3D-printer section. 

**Figure 4:**
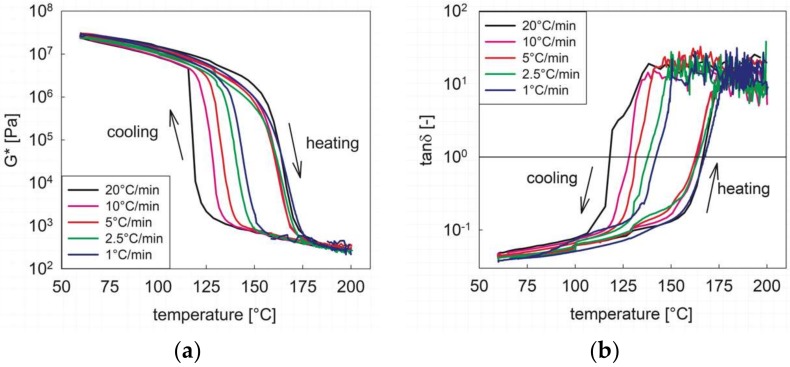
The viscoelastic response during the cooling/heating cycle at different temperature rates of change. (**a**) complex modulus; (**b**) loss factor.

In all processes involving a thermally induced phase change, the transitional phenomena depend on the temperature rate of change, as both thermodynamic and kinetic effects are present. Figure 4 shows the cooling/heating cycle (the holding phase data at 60 °C have been omitted to improve the clarity) at different rates, from 20 to 1 °C/min. The complex modulus, G* (Figure 4a), shows all the features already discussed above. At the highest rate of change (20 °C/min), the onset of phase transition upon cooling takes place to the lowest temperature. As the rate decreases, the transition takes place at increasingly higher temperatures, indicating that the phase transition kinetics upon cooling are becoming significantly less relevant compared to thermodynamic effects [31]. On the contrary, the dependence of the melting behavior upon the heating rate is not relevant, as the curves essentially superimpose and no clear trend is observed. It is also apparent that, at any cooling/heating rate, the sol-to-gel transition realized upon cooling the melt is much “sharper” than the corresponding melting process, in agreement with the calorimetric data shown in Figure 1. It can be deduced that the formation of the microdomains is a more difficult process than their symmetrical melting, thus giving more strength to the hypothesis that melting is determined by a progressive erosion of the hard phase domains, with a gradual loss of connectivity [30].

A more quantitative estimate of the transition temperatures during both cooling and heating can be done with the help of Figure 4b, which shows the loss factor, that is, the ratio between the loss and elastic components of the viscoelastic modulus. The scattering of the data at high temperatures, which is due to the elastic component below or at the boundary of the measurable instrument sensitivity (see above, Figure 1), does not significantly affect the following analysis. According to the empirical definition of the critical gel point given above, a “rheological” phase transition temperature can be located where the two moduli become equal, i.e., when tanδ = 1. The crystallization and melting temperatures as derived from the above criterion are reported in Table 1 for the five cooling/heating rates used. It is confirmed that the two temperatures get closer as the rate decreases and that melting is less sensitive than crystallization to the thermal rate of change. It is also interesting to compare the rheological results of Table 1 with those obtained by DSC as reported in Figure 1. At the same rate of 10 °C/min, the melting rheological temperature is slightly higher than the peak DSC value. Conversely, upon cooling, the sol/gel transition temperature is significantly higher than the peak DSC one. This result is not surprising at all, since the two measurements have completely different meanings. *T*_m_ and *T*_c_ derived from calorimetry merely correspond to the peak in the heat flow curves and, as such, to the maximum phase transition rate. Conversely, their rheological counterparts give a mechanical information, namely, the transition from a liquid-like to a solid-like behavior. The physical consequence is that the critical gel state, where the liquid response gives room to the solid behavior, is reached after the most part of the microstructure has been already formed. It must be noticed that this not always true. While the same behavior has been already observed for other segmented block copolymers like TPU [29,30], a critical gel state reached during the early phase transition stages is often observed in semi-crystalline polymers [32], indicating that the nature of the microstructure (microdomains in the first case, crystallites embedded in the amorphous matrix in the second one) strongly affects the mechanical quality of the transitioning material.

### 3.3. Isothermal Rheology

The non-isothermal rheological measurements indicate that the process of microphase separation upon cooling of PEOT/PBT begins at relatively high temperatures (at least 155 °C, see for example Figure 4a for the cooling rate of 1 °C/min) and proceeds even at much lower temperatures, thus spanning a wide thermal range. This suggests that, in order to better explore the role of temperature on the sol/gel (or disorder-to-order) transition process, isothermal experiments can be particularly useful.

**Figure 5:**
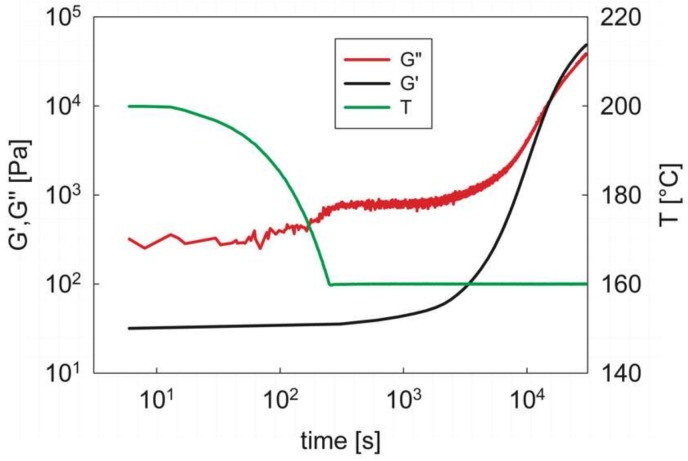
The elastic and loss modulus as a function of time in an isothermal experiment at 160 °C. The temperature history is also reported on the right *y*-axis. *G*′ data have been smoothed for better clarity.

The results of a typical isothermal test are reported in Figure 5. Here, after sample loading at 200 °C, the temperature is rapidly dropped to the test temperature (in this case 160 °C) by a cooling ramp at 20 °C/min. When the target temperature is reached, the temporal evolution of the viscoelastic moduli at a fixed frequency is monitored. Notice that the G′ data have been smoothed, due to the high temperature data scattering (see above), simply to allow for a better reading of the results.

In the first part of the experiment the rapid cooling only determines a slight increase of the viscoelastic moduli, typical of a polymer melt. The melt state is maintained also when the target temperature is reached. Only after an extra time at the constant temperature has elapsed (in this case, of the order of a few thousand seconds) the moduli start to increase relevantly, indicating the onset of the microphase separation. As in the non-isothermal case, the cross-over between the viscoelastic moduli indicates the critical gel point and the transition from liquid-like to solid-like behavior. At longer times, the moduli versus time curves are seen to bend towards a horizontal plateau, which is not reached in this particular case due to the extremely long experimental times.

The results of Figure 5 clearly indicate the importance of the kinetics and of their temperature dependence on the microphase separation. Inspection of Figure 4, for example, indicates that even at the lowest cooling rate (1 °C/min) the polymer is still in its melt state down to a temperature of about 160 °C. Two important consequences derive: first, even at a temperature as high as 160 °C the system presents a disorder-to-order transition; second, at these high temperatures the phase transition kinetics are relatively slow, as confirmed by the long “induction time”, that is, the time required to see an appreciable increase of the viscoelastic moduli.

**Figure 6:**
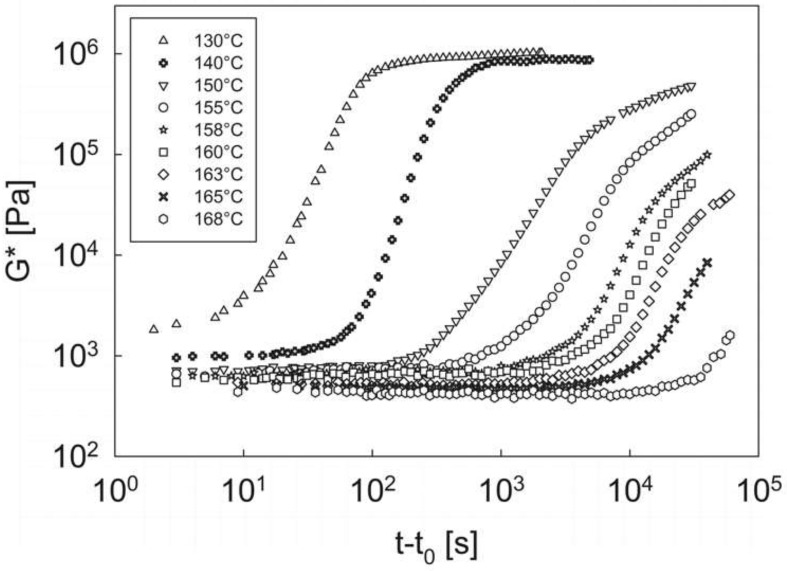
The complex modulus as a function of the reduced time *t* − *t*_0_ (where *t*_0_ is the time when the target temperature is first reached) during isothermal experiments at different annealing temperatures.

As in all temperature-driven phase transition phenomena, including crystallization and phase separation, the kinetics are expected to become faster as the temperature is decreased. This is confirmed in Figure 6, where the isothermal evolution of the complex modulus is plotted as a function of time at different annealing temperatures. The experimental protocol is the same as that of Figure 6. In this case, however, the complex modulus is plotted as a function of the reduced time, *t* − *t*_0_, where *t*_0_ is the time when the target temperature is first reached.

The message of Figure 6 is very clear: the kinetics of the phase transition process become faster as the temperature decreases, thus determining an earlier increase in the complex modulus evolution. Furthermore, although the long-time tail at the higher temperatures cannot be always captured due to the phase transition kinetics being too slow with respect to the experimentally affordable time window, the system seems to tend to an equilibrium value of the modulus. It is important to underline that, above the temperature of 168 °C (the highest temperature reported in Figure 6) no hint of phase transition is detected, at least for experimental times as long as about 10^5^ s.

From the analysis of Figure 5 and Figure 6 it can be concluded that, for a given temperature, the system evolves from a melt, homogenous state, to an equilibrium, microphase-separated morphology, as the soft and hard domains progressively form, grow, and stabilize with time, passing through the sol/gel transition. The kinetics of this process can be quantified by determining, at each temperature, the critical gelation reduced time, (*t* − *t*_0_)_cg_, that is the time corresponding to the cross-over of the viscoelastic moduli (see above). The values of the reduced critical gelation time at the different annealing temperatures, are reported in Table 2. The same table reports also the reduced induction time, i.e., the characteristic time necessary to appreciate a change in the viscoelastic properties after the beginning of the transition process. Among the various definitions of the induction time found in the literature [33,34] we decide to calculate it as the time necessary for the complex modulus to double its value with respect to the initial one [35]. Notice that the induction time cannot be measured at the lowest temperature (130 °C) because, as it is apparent from Figure 6, the microphase separation process has already started when the annealing temperature has been just reached. Conversely, at the highest temperature (168 °C) the critical gelation time cannot be appreciated as it goes well beyond the affordable experimental time window.

It is obvious that the progressive slowing down of the phase transition kinetics as the temperature increases cannot be extended to any arbitrary temperature. As already mentioned in the Introduction, there is a limiting temperature above which the repulsive interactions between hard segments and soft segments, which are responsible for the phase separation, are not strong enough to overcome the randomizing action of thermal motion. Such a randomizing action determines the formation of a single-phase homogeneous melt [36]. The order–disorder transition (ODT) temperature is also an important technological parameter. It is the temperature above which the material will never be able to solidify, no matter how much time is allowed the system to undergo the phase transition.

**Figure 7:**
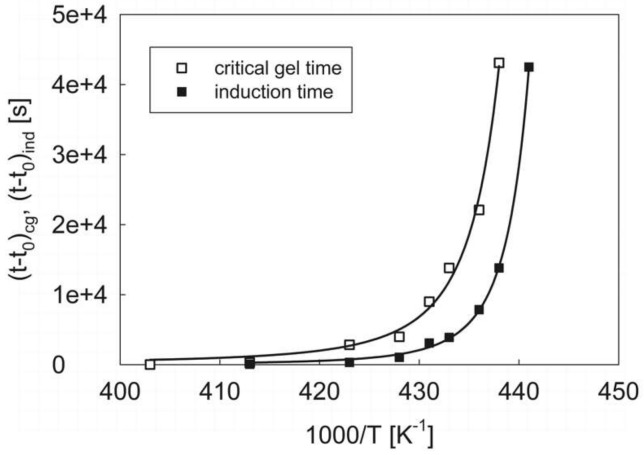
The reduced critical gel and induction times as a function of the absolute temperature. The solid lines are the non-linear regression fits of Equation (2).

A rheological estimate of the ODT temperature can be done by using the experimental results of the isothermal measurements. Along the same lines describing the kinetics of the polymer crystallization kinetics [37], we can assume that the characteristic time for the phase transition follows a law of the type:

(2) $$t=A\exp\left[\frac{B}{T\left(T_{\mathrm{ODT}}-T\right)}\right]$$

where *t* can be either the critical gel or the induction time, *A* is a numerical pre-factor, *B* is a pseudo-activation energy, *T*_ODT_ is the order-to-disorder temperature and *T* is the test temperature. The non-linear regression of Equation (2) to the data of Table 2 is reported in Figure 7, where the reduced characteristic times are both plotted as a function of the absolute temperature. The sets of parameters obtained from the regression procedure are listed in Table 3.

The agreement between experimental data and model prediction is very good, especially in the high temperature region, that is, much above the glass transition temperature of the amorphous phase, where the crystallization kinetics model expressed by equation is expected to hold. In particular, Table 3 shows that the *T*_ODT_ parameter obtained by the two different sets of data is practically the same. This fact, along with the similar values found for the pseudo-activation energy *B*, confirms that the temperature dependence of the two characteristic times is the same, as they are two faces of the same physical process. Moreover, the consistency between critical gel time and induction time correlations allows for a robust determination of the ODT temperature, which can be situated around 187 °C. This means that, above such a temperature, the system is expected to live in a fully homogeneous, not phase-separated melt state.

## 4. Concluding Remarks

In this work the phase transition behavior of a PEOT/PBT segmented copolymer has been deeply investigated by rheological techniques. It has been shown that rheology can accurately describe the kinetics and the thermodynamics of the microphase separation induced by temperature changes. At the same time, since rheology probes the mechanical response both in the liquid and in the intermediate, soft-matter state, the quantitative information collected from the measurements constitutes a very useful tool for the design and the implementation of the manufacturing process, in this case the 3D-printing bio-scaffold fabrication. In fact, at least from the processing point of view, the polymer must possess well defined rheological properties. On the one hand, the injection section of the 3D-printer calls for a low viscosity in the melt state. This quality is necessary to determine low injection pressures, which is important to minimize the stress applied to the cell population, but also to reduce the mechanical requirements of the system [38]. On the other hand, in the filament deposition phase of the process, the transition from liquid-like to solid-like behavior is crucial, as the adhesion between layers is favored by a relatively slow kinetics of the phase transition, whereas the structural consistency and the dimensional stability require a sufficiently fast kinetics. As a consequence, the rheology of the phase sol/gel transition, and its dependence upon the thermal history, plays a crucial role. Based on the above considerations, the following main conclusions can be drawn:

• non-isothermal experiments in the melt state allowed to determine the viscosity of PEOT/PBT in the liquid state and its quantitative dependence upon temperature. The material exhibited sufficiently low viscosities, ranging between about 30 and 100 Pa s in the temperature range 200–140 °C, with a relatively modest temperature dependence;
• non-isothermal experiments at lower temperatures indicate that the sol/gel transition upon cooling is strongly affected by the thermal history, while the same does not hold in the symmetrical melting stages. Comparing rheological and calorimetric data also indicates that the transition from a liquid-like to a solid-like material takes place when the most part of the microphase separation has been completed, suggesting that the actual solidification is due to a rearrangement of the microstructure, rather than the mere phase transition. From the processing viewpoint, the results from the non-isothermal rheometry are extremely useful. The significant shift of the solidification to lower temperatures as the cooling rate increases provides relevant information to design both the characteristic time and the geometrical patterns of the filament deposition and cooling stages of the process. A too-high cooling rate would move the solidification to shorter times, but at the same time it would hinder a proper adhesion between two filaments deposited on successive layers. A too-low cooling rate would be detrimental to the dimensional stability of the scaffolds;
• the isothermal rheology during phase transition, which allowed also the determination of a thermodynamic ODT temperature, suggests a different way to manage the above compromise between solid-like (bio-scaffold consistency) and liquid-like (inter-filament adhesion) behavior. Running the filament deposition stages at a suitable, higher than ambient, constant temperature, would allow to easily find the above compromise. Indeed, as indicated by Figure 6, Table 3 and Equation (2), a proper choice of the isothermal phase transition temperature determines a specific characteristic time for the phase transition. This, in turn, becomes a processing parameter, to be compared with the characteristic time of filament deposition in order to get the best 3D-printing conditions.

In summary, it is confirmed that rheology plays an important role as a two-face experimental tool, able to help in determining the polymer microstructure during phase transition, and providing crucial information on the processing aspects of the manufacturing stages.

## Figures and Tables

**Table 1:** The rheological crystallization and melting temperature (for the definition see the text) at different cooling/heating rates.

Cooling/Heating Rate (°C/min)	*T*_c,rheol_ (°C)	*T*_m,rheol_ (°C)
20	121	166
10	128	163
5	132	164
2.5	138	165
1	142	167

**Table 2:** The critical gelation and the induction reduced times at different annealing temperatures.

Annealing Temperature (°C)	(*t* − *t*_0_)_cg_ (s)	(*t* − *t*_0_)_ind_ (s)
130	25	–
140	288	68
150	2840	325
155	3980	1050
158	8990	3090
160	13,800	3900
163	22,100	7850
165	43,100	13,800
168	>60,000	42,500

**Table 3:** The fitting parameters of Equation (2) for the reduced critical gelation and induction times.

Characteristic Time	*A* (s)	*B* × 10^−4^ (K^2^)	*T*_ODT_ (K)
(*t* − *t*_0_)_cg_	41.1	6.35	459
(*t* − *t*_0_)_ind_	5.50	7.94	461
